# Is early-follicular long-acting GnRH agonist protocol an alternative for patients with polycystic ovary syndrome undergoing in vitro fertilization?

**DOI:** 10.1186/s12958-022-01007-z

**Published:** 2022-09-10

**Authors:** Di Wang, Ting Chu, Ting Yu, Jun Zhai

**Affiliations:** 1 Center for Reproductive Medicine, The First Affiliated Hospital of Zhengzhou University, Zhengzhou, 450000 Henan China; 2 Henan Key Laboratory of Reproduction and Genetics, The First Affiliated Hospital of Zhengzhou University, Zhengzhou, Henan China; 3 Henan Provincial Obstetrical and Gynecological Diseases (Reproductive Medicine) Clinical Research Center, The First Affiliated Hospital of Zhengzhou University, Zhengzhou, Henan China

**Keywords:** Polycystic ovary syndrome, Early-follicular long-acting GnRH agonist long protocol, Midluteal short-acting GnRH agonist long protocol, In vitro fertilization, Embryo transfer, Perinatal complications

## Abstract

**Background:**

We aimed to compare the clinical and perinatal outcomes of patients with polycystic ovary syndrome (PCOS) undergoing in vitro fertilization/intracytoplasmic sperm injection (IVF/ICSI) treatment with either an early-follicular long-acting gonadotropin-releasing hormone agonist (GnRH-a) long protocol (EFLL) or a midluteal short-acting GnRH-a long protocol (MLSL).

**Methods:**

This single–center, retrospective study, included patients with PCOS who underwent IVF/ICSI from January 2013 to June 2019 at the First Affiliated Hospital of Zhengzhou University, Zhengzhou, China. Patients underwent either MLSL (1179 cycles) or EFLL (2390 cycles). The primary outcomes were pregnancy outcomes, perinatal and maternal complications.

**Results:**

Fresh embryo transfer (59.12% vs. 55.47%, *P* = 0.038), clinical pregnancy (75.23% vs. 53.82%, *P* = 0.001), and live birth rates (63.27% vs. 42.05%, *P* = 0.010) were higher in the EFLL group. However, the proportion of patients “freezing all" for high risk of ovarian hyperstimulation syndrome (OHSS) (24.27% vs. 32.06%, *P* = 0.001) and ectopic pregnancy (1.51% vs. 5.97%, *P* = 0.002) were lower in the EFLL group than in the MLSL group. The incidence of gestational diabetes was higher in the EFLL group than in the MLSL group (5.08% vs. 1.42%, RR 3.714, 95% confidence interval (CI) 1.474–9.360, *P* = 0.003). There were no significant differences in the incidence of hypertension, premature rupture of membranes, placenta previa, congenital heart disease, or neonatal weight between the two groups. Logistic regression results showed that age (OR 0.966, 95% CI 0.941–0.993, *P* = 0.013), treatments (OR 2.380, 95% CI 1.833–3.089, *P* = 0.001), and endometrial thickness on trigger day (OR 1.115, 95% CI 1.070–1.162, *P* = 0.001) were correlated with clinical pregnancy. Pre-pregnancy BMI (OR 1.098, 95% CI 1.002–1.204, *P* = 0.046), fasting plasma glucose (FPG) (OR 3.096, 95% CI 1.900–5.046, *P* = 0.001), and treatments (OR 3.458, 95% CI 1.359–8.800, *P* = 0.009) were correlated with gestational diabetes mellitus (GDM). Treatments (OR 0.291, 95% CI 0.148–0.575, *P* = 0.001) and endometrial thickness on trigger day (OR 0.834, 95% CI 0.722–0.962, *P* = 0.013) were correlated with ectopic pregnancy.

**Conclusion:**

The early-follicular long-acting GnRH agonist long protocol can be used as an ideal assisted reproductive technology (ART) pregnancy assistance program for patients with PCOS, but obese patients should be encouraged to lose weight before ART treatments to reduce the risk of GDM.

**Supplementary Information:**

The online version contains supplementary material available at 10.1186/s12958-022-01007-z.

## Background

Polycystic ovary syndrome (PCOS) is a common reproductive, endocrine, and metabolic disease, with an incidence of approximately 9%–18% in women of reproductive age [1]. It can manifest as abnormal menstruation, infertility, hyperandrogenism, and polycystic appearing ovaries on ultrasound. The condition can also be accompanied by metabolic abnormalities such as obesity, insulin resistance, and dyslipidemia. Most patients with PCOS present with insulin resistance and compensatory hyperinsulinemia, leading to a higher risk of type 2 diabetes, gestational diabetes mellitus (GDM), and other pregnancy-related complications [2]. An increasing number of infertile patients with PCOS choose to use assisted reproductive technology (ART) to conceive. Patients with PCOS are also at high risk of ovarian hyperstimulation syndrome (OHSS). In order to reduce the risk of OHSS, a gonadotropin-releasing hormone (GnRH) antagonist protocol is often used as the first choice for controlled ovarian stimulation in patients with PCOS. A previous study showed that in the normal population, the cumulative live birth rates of GnRH antagonist protocol, early-follicular long-acting GnRH agonist long protocol (EFLL), and midluteal short-acting GnRH agonist long protocol (MLSL) were not statistically significant (71.4% vs. 75.5% vs. 72.2%, respectively), but the live birth rate of fresh embryo transfer cycles was significantly higher in EFLL than in MLSL and GnRH antagonist protocol (62.6% vs. 52.1% vs. 45.6%, respectively) [3]. Chen et al. applied a comparative proteomics analysis and found that GnRH antagonists were more harmful to endometrial receptivity than agonists [4]. Previously, EFLL has been widely used due to its advantages of higher clinical pregnancy rate and lower risk of OHSS. However, few studies have discussed the effect of EFLL in patients with PCOS. This study retrospectively analyzed the clinical data of patients with PCOS, who underwent fresh in vitro fertilization/intracytoplasmic sperm injection (IVF/ICSI) using EFLL or MLSL, to compare the effects of different ovulation induction protocols on pregnancy outcomes and perinatal maternal and fetal complications.

## Methods

This retrospective cohort study was approved by the Ethics Review Committee of the First Affiliated Hospital of Zhengzhou University. Written informed consent was waived due to the retrospective nature of the study. This analysis was conducted on patients with PCOS who underwent the first cycle of IVF/ICSI-assisted pregnancy treatment using either EFLL or MLSL at the Center of Reproduction Medicine of the First Affiliated Hospital of Zhengzhou University from January 2013 to June 2019. The inclusion criteria were as follows: ① aged 20–40 years and ② diagnosed with PCOS (in line with Rotterdam PCOS diagnostic criteria in 2003). The exclusion criteria were: ① untreated hydrosalpinx; ② endocrine diseases such as hyperprolactinemia and diabetes mellitus; ③ uterine malformation; ④ preimplantation genetic diagnosis/preimplantation genetic screening; ⑤ uterine fibroids with a diameter of > 3 cm and compression of the endometrium; ⑥ patients with endometriosis/adenomyosis; ⑦ intrauterine adhesion; ⑧ cervical insufficiency; and ⑨ history of tuberculosis.

### Ovulation induction program and embryo transfer

#### Early-follicular long-acting GnRH agonist long protocol

Patients were administered 3.75 mg of the long-acting gonadotropin-releasing hormone agonist (GnRH-a), triptorelin (Pfizer Pharmaceutical Co., Ltd., Germany), on the 2nd to 3rd day of spontaneous menstruation or menstruation after oral administration of Dydrogesterone (Abbott Healthcare Products B.V.). Serum follicle-stimulating hormone (FSH), luteinizing hormone (LH), estrogen (E2), and progesterone (P) were measured 28 days after injection. Follicle size was monitored using transvaginal ultrasound.

#### Midluteal short-acting GnRH agonist long protocol

A short-acting GnRH-a, tripraline (IPSEN Biotechnology, France), was administered subcutaneously at a dose of 0.1 mg per day for 14 to 16 days, starting on the 21^st^ or 22nd day of spontaneous menstruation or the 16th day of oral administration of drospirenone and ethinylestradiol tablets (Bayer AG, Germany) at a dose of 1 tablet per day. Serum FSH, LH, E2, and P were measured after injection, meanwhile follicular size was monitored by vaginal ultrasound.

Gonadotropin (Gn) was administered for controlled ovarian hyperstimulation when the downregulation standards were reached (FSH < 5 U/L, LH < 3 U/L, E2 < 50 pg/mL, antral follicle diameter about 3–5 mm, no ovarian cysts > 10 mm). The dosage of Gn in both protocols depended on factors such as the patients’ age, basal antral follicle count (AFC), body mass index (BMI), and basal hormone level. The dosage was adjusted according to the size of the follicles and the levels of FSH, LH, E2, and P.

When at least 60% of the follicles were > 16 mm in diameter or when at least three dominant follicles were ≥ 18 mm, 250ug of Azer (Merck Serono, Darmstadt, Germany) and 2000 IU of human chorionic gonadotrophin (hCG) (Livzon Pharmaceutical, China) were subcutaneously injected on the same night to trigger ovulation. About 36–37 h after the trigger, transvaginal ultrasound-guided puncture was performed for oocyte retrieval. The method of fertilization, IVF/ICSI, was based on semen quality. Fresh embryo transfers were performed 3–5 days after oocyte retrieval under ultrasound guidance based on embryo quality and patients’ overall and endometrial conditions. The transfer was cancelled if patients were deemed to be at high risk for OHSS or a uterine effusion was demonstrated. The luteal supplementation protocol was started from the day of oocyte retrieval. Progesterone sustained-release vaginal gel (Crinone, Merck Serono, Germany) and dydrogesterone tablets (Duphaston, Abbott, Labora Netherlands) were added at the dosage of 90 mg per day and 20 mg po BID respectively.

### Outcome and follow-up

To follow up with the pregnancy outcomes, we drew serum B-hCG levels 14 days after embryo transfer. Clinical pregnancy was confirmed when a gestational sac could be seen on ultrasound examination at 35 days after transplantation. Fetal nuchal translucency examinations were performed 9–10 weeks after embryo transfer. During the perinatal period, trained nurses provided follow-up via telephone. Standardized questionnaires were used to collect information on perinatal complications, gestational age, mode of delivery, neonatal sex, birth weight, diseases among newborns, and treatments. The follow-up information was recorded in detail and stored in the electronic medical records. The research data were extracted from the electronic database of our hospital.

### Observation indicators

General patient information, days and total dosage of Gn, number of embryos transferred, hormone levels on day of trigger, incidence of OHSS, clinical pregnancy rate, spontaneous abortion rate, ectopic pregnancy rate, and perinatal maternal and infant adverse outcomes rates were recorded. The following metrics were also assessed: The grading criteria of high-quality embryos were based on previous publications of our center [5]. High-quality embryos include grades I and II.; high risk of OHSS was defined when the E2 level was > 3000 pg/mL on trigger day and > 15 oocytes were retrieved [6]; clinical pregnancy rate = number of clinical pregnancy cycles/number of transfer cycles × 100% [7]; live birth rate = number of live birth cycles/number of transfer cycles × 100%; preterm birth rate = number of premature delivery cycles/number of transfer cycles × 100% [8]; spontaneous abortion rate = number of spontaneous abortion cycles/number of clinical pregnancy cycles × 100% [9]; and ectopic pregnancy rate = number of ectopic pregnancy cycles/number of clinical pregnancy cycles × 100%.

### Statistical methods

All data in this study were analyzed and calculated using SPSS. Continuous variables are expressed as mean ± standard deviation (x ± s) and categorical variables are expressed as percentages. The t-test was used to determine the significance of the difference between the mean values of two continuous variables. The Chi-square (χ^2^) test was used to assess differences in the proportion of categorical variables between two or more groups. Logistic regression analysis was conducted on the factors affecting the occurrence of pregnancy complications. Statistical significance was established at *P* < 0.05.

### Results

#### Baseline comparison of patients

A total of 3569 patients were included in this study, which included 1179 cycles using MLSL and 2390 cycles using EFLL. A comparison of the patients’ baseline characteristics is shown in Table 1. There were no statistically significant differences in the patients’ age, BMI, duration of infertility, basal hormone levels, or fasting plasma glucose.

**Table 1:** Baseline comparison of patients

	MLSL	EFLL	*P* value
	*n* = 1179	*N* = 2390	/
Age (year)	28.883 ± 3.872	28.722 ± 3.752	0.239
Duration of infertility (year)	4.279 ± 2.747	4.146 ± 2.760	0.184
BMI (kg/m^2^)	24.330 ± 3.633	24.263 ± 3.448	0.595
LH (mIU/ml)	9.957 ± 6.655	9.630 ± 7.079	0.185
LH/FSH	1.718 ± 1.132	1.686 ± 1.152	0.433
E2(pg/ml)	41.457 ± 18.726	40.257 ± 17.760	0.064
T(ng/ml)	1.477 ± 7.125	1.081 ± 6.167	0.105
AMH (ng/ml)	7.764 ± 4.441	7.955 ± 4.381	0.539
FPG (mmol/l)	4.836 ± 0.445	4.859 ± 0.474	0.290
No. of cleavage transfer cycles (n)	*n* = 514	*n* = 1032	0.779
1	17	37	
2	497	995	
No. of blastocyst transfer cycles (n)	*n* = 140	*n* = 381	0.800
1	139	379	
2	1	2	

Note: Continuous data: mean ± SD. Categorical data: % (n/N)

*BMI* body mass index, *HCG* human chorionic gonadotropin, *LH* luteinizing hormone, *FSH* follicle-stimulating hormone, *AMH* anti-Müllerian hormone, *FPG* fasting plasma glucose

### Comparison of laboratory parameters and clinical outcomes

The number of transferable embryos (5.83 ± 3.42 vs. 6.56 ± 4.00) and the high-quality embryo rate (54.23% vs. 59.37%) in the EFLL group were lower than in the MLSL group (*P* < 0.05), while the rate of fresh embryo transfer cycles (59.12% vs. 55.47%), the rates of biochemical pregnancy (79.23% vs. 58.87%), clinical pregnancy (75.23% vs. 53.82%), live birth (63.27% vs. 42.05%) and full-term birth (50.88% vs. 32.87%) were higher than in the MLSL group (*P* < 0.05). The proportion of patients “freezing all” for high risk of OHSS (24.27% vs. 32.06%) was lower than in the MLSL group. There were no statistically significant differences in the abortions, premature births, or incidence of moderate to severe OHSS between the two groups (*P* ≥ 0.05; Table 2).

**Table 2:** Comparison of laboratory parameters and clinical outcomes

	MLSL	EFLL	***P*** value
Length of stimulation (d)	11.894 ± 2.139	14.725 ± 2.660	< 0.001
Total dosage of Gn (IU)	1666.979 ± 706.009	2263.929 ± 909.607	< 0.001
E2 on trigger day (pg/ml)	6360.655 ± 3283.401	3992.922 ± 2227.219	< 0.001
LH on trigger day (mIU/ml)	1.446 ± 0.693	0.738 ± 0.957	< 0.001
Endometrial thickness on trigger day (mm)	11.240 ± 2.554	12.134 ± 2.360	< 0.001
No. of oocytes retrieved (n)	16.906 ± 7.813	18.078 ± 7.978	< 0.001
No. of 2PN (n)	10.905 ± 5.987	10.852 ± 5.963	0.804
No. of transferable embryos	6.559 ± 4.004	5.832 ± 3.415	< 0.001
High-quality embryos rate (%)	59.37 (7531/12684)	54.23 (13892/25619)	< 0.001
Moderate to severe OHSS rate (%)	2.46 (29/1179)	3.22 (77/2390)	0.207
“Freezing all” for high risk of OHSS (%)	32.06 (378/1179)	24.27 (580/2390)	< 0.001
Implantation rate (%)	37.85 (436/1152)	56.76 (1368/2410)	< 0.001
Biochemical pregnancy rate (%)	58.87 (385/654)	79.23 (1120/1413)	< 0.001
Clinical pregnancy rate (%)	53.82 (352/654)	75.23 (1063/1413)	< 0.001
Live birth rate (%)	42.05 (275/654)	63.27 (894/1413)	0.010
Full-term birth rate (%)	32.87 (215/654)	50.88 (719/1413)	< 0.001
Spontaneous abortion rate (%)	8.26 (54/654)	10.83 (153/1413)	0.070
PTB rate (%)	8.10 (53/654)	11.11 (157/1413)	0.518

Note: Categorical data: % (n/N)

*Gn* gonadotropin, *LH* luteinizing hormone, *OHSS* ovarian hyperstimulation syndrome, *PTB* preterm birth

### Comparison of maternal and fetal complications during pregnancy and the perinatal period

There were a total of 1415 cycles of clinical pregnancy in the two groups, including 352 cycles in the MLSL group and 1063 cycles in the EFLL group. The rate of gestational diabetes in the EFLL group was higher than that in the MLSL group (*P* < 0.05), while the ectopic pregnancy rate was lower than that in the MLSL group (*P* < 0.05). There were no statistical differences in obstetric complications such as preeclampsia, multiple birth rate, placenta previa, premature rupture of membranes, and neonatal birth between the two groups (Table 3).

**Table 3:** Comparison of maternal and fetal complications during pregnancy and the perinatal period

	MLSL	EFLL	RR (95% CI)	*P* value
	*n* = 352	*n* = 1063		
PE	12/352 (3.41)	57/1063 (5.36)	1.605 (0.851,3.028)	0.140
ICP	0	4/1063 (0.38)	1.004 (1.000,1.007)	0.566
IDA	1/352 (0.28)	0	0.997 (0.992,1.003)	0.561
GDM	5/352 (1.42)	54/1063 (5.08)	3.714 (1.474,9.360)	0.003
Ectopic pregnancy	21/352 (5.97)	16/1063 (1.51)	0.241 (0.124,0.467)	0.002
Multiple pregnancy	99/352 (28.13)	280/1063 (26.34)	0.914 (0.698,1.196)	0.512
Placenta previa	2/352 (0.57)	2/1063 (0.19)	0.330 (0.046,2.351)	0.559
PROM	10/352 (2.84)	51/1063 (4.79)	1.724 (0.865,3.432)	0.117
Cesarean	212/352 (60.23)	681/1063 (64.06)	1.177 (0.919,1.508)	0.196
Gestational age at birth	37.920 ± 2.350	37.750 ± 2.271	/	0.269
Macrosomia	28/340 (8.24)	100/1095 (9.13)	1.120 (0.723,1.735)	0.612
LBW	80/340 (23.53)	264/1095 (24.11)	1.032 (0.775,1.375)	0.827
Malformation	3/340 (0.88)	9/1095 (0.82)	0.931 (0.251,3.458)	1.000
CHD	1/340 (2.9)	6/1095 (0.55)	1.868 (0.224,15.569)	0.832

Note: Categorical data: n/N (%);

*PE* preeclampsia, *ICP* intrahepatic cholestasis of pregnancy, *IDA* iron-deficiency anemia, *GDM* gestational diabetes, *PROM* premature rupture of membranes, *LBW* low birth weight, *CDH* Congenital Heart Disease

### Logistic regression

**Fig. 1:**
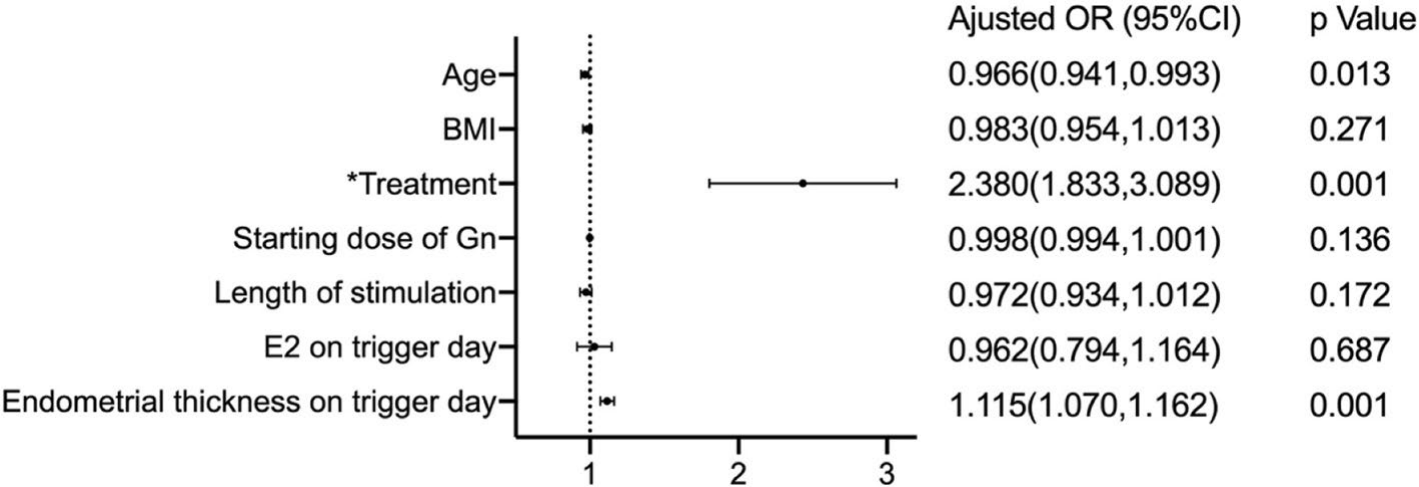
Forest plot: multivariate logistic regression performed for clinical pregnancy

**Fig. 2:**
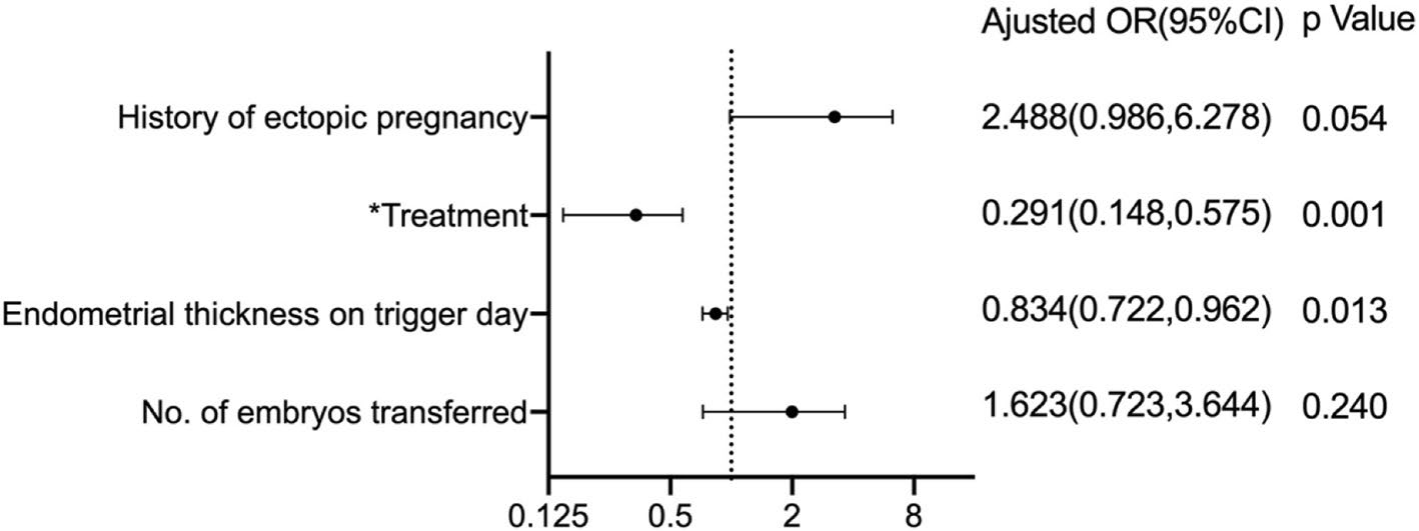
Forest plot: multivariate logistic regression performed for ectopic pregnancy. * Both of them used MLSL as a control

Univariate logistic regression was performed for variables that may affect the occurrence of clinical pregnancy and ectopic pregnancy, and multivariate logistic regression was performed again for variables which were found to be significant following univariate logistic regression. Age, BMI, treatment, starting dose of Gn, length of stimulation, E2 on trigger day and endometrial thickness on trigger day were included for the multivariate logistic regression for clinical pregnancy. History of ectopic pregnancy, treatment, endometrial thickness on trigger day and number of embryos transferred were included for the multivariate logistic regression for ectopic pregnancy. The results are shown in Tables 4 and 5 and Figs. 1 and 2.

**Table 4:** Logistic regression. Univariate logistic regression performed for clinical pregnancy

	non-clinical pregnancy	clinical pregnancy	OR (95% CI)	*P*
Age	29.350 ± 4.067	28.730 ± 3.687	0.959(0.926,0.982)	0.001
BMI	24.717 ± 3.456	24.265 ± 3.539	0.964(0.939,0.990)	0.007
Treatment				
MLSL	303/653(46.40)	351/1414(24.82)	Reference	-
EFLL	350/653(53.60)	1063/1414(75.18)	2.622(2.156,3.188)	0.001
Starting dose of Gn	128.407 ± 40.798	118.494 ± 31.486	0.992(0.990,0.995)	0.001
Length of stimulation	13.480 ± 2.874	13.830 ± 2.777	1.047(1.012,1.082)	0.008
Total dosage of Gn	2141.720 ± 951.762	2123.784 ± 926.781	1.000(1.000,1.000)	0.685
No. of oocytes retrieved	14.730 ± 6.194	14.650 ± 5.781	0.998(0.982,1.013)	0.766
E2 on trigger day	4091.179 ± 2318.508	3703.734 ± 2003.550	0.780(0.660,0.923)	0.004
Endometrial thickness on trigger day	11.278 ± 2.467	12.092 ± 2.371	1.153(1.108,1.200)	0.001

**Table 5:** Logistic regression***.*** Univariate logistic regression performed for ectopic pregnancy

	non ectopic pregnancy	ectopic pregnancy	OR(95%CI)	*P* value
Age	28.740 ± 3.691	28.270 ± 3.517	0.961 (0.880,1.057)	0.442
Type of infertility				
Primary infertility	917/1377 (66.59)	23/37 (62.16)	Reference	-
Secondary infertility	460/1377 (33.41)	14/37 (37.84)	1.215 (0.619,2.383)	0.571
History of ectopic pregnancy	92/1377 (6.68)	6/37 (16.22)	2.705 (1.101,6.650)	0.030
No. of abortions	0.300 ± 0.631	0.380 ± 0.639	1.188 (0.759,1.861)	0.451
Fertilization method				
IVF	1093/1377 (79.38)	32/37 (86.49)	Reference	-
ICSI	284/1377 (20.62)	5/37 (13.51)	1.661 (0.642,4.302)	0.296
Treatment				
MLSL	330/1377 (23.97)	21/37 (56.76)	Reference	-
EFLL	1047/1377 (76.03)	16/37 (43.24)	0.241 (0.124,0.467)	0.001
Moderate to severe OHSS	67/137 (4.87)	1/37 (2.70)	0.544 (0.073,4.025)	0.551
Endometrial thickness on trigger day (mm)	12.121 ± 2.358	10.946 ± 2.573	0.804 (0.696,0.928)	0.003
No. of embryos transferred	1.730 ± 0.453	1.860 ± 0.419	2.165 (0.909,5.156)	0.081

## Discussion

We performed a comprehensive and retrospective analysis of the laboratory and clinical outcomes of patients with PCOS who underwent fresh IVF/ICSI using EFLL or MLSL. The number of oocytes retrieved and the cycle rate of fresh embryo transfer in EFLL group were higher than those in MLSL group, but the proportion of patients at high risk of OHSS was lower than that in MLSL group. Although the high-quality embryo rate and the number of transferable embryos were lower in the MLSL group, the clinical pregnancy rate and live birth rate were higher in the EFLL group. A previous study found that in fresh transplantation cycles of patients with PCOS, the number of oocytes retrieved (17.49 ± 7.17 vs. 12.85 ± 7.26) and the clinical pregnancy rate (53.92% vs. 40.00%) were significantly increased in EFLL compared with the GnRH antagonist protocol, and there was no significant difference in the incidence of OHSS [10]. The incidence of moderate to severe OHSS was not statistically different between two groups in our study, but it was significantly lower than that in previously published studies [11–14]. These results suggest that PCOS patients who undergo EFLL have more chances to transfer a fresh embryo and a higher pregnancy rate without increasing the occurrence of OHSS, and the time to reach pregnancy is shortened.

Patients with PCOS often have reduced endometrial receptivity due to their impaired glucose metabolism and abnormal hormone levels [15–18]. The application of long-acting GnRH-a facilitates the recovery of the endometrial glands [19] and can improve endometrial receptivity [20]. Meanwhile, some studies have found that as endometrial thickness increases, the pregnancy rate also increases [21, 22]. We found that the endometrial thickness on the day of trigger was greater in the EFLL group than in the MLSL group (12.134 vs. 11.240). Logistic regression indicated that age, endometrial thickness and treatment were correlated with clinical pregnancy, which suggests that EFLL may be more conducive to embryo implantation by increasing endometrial thickness and improving endometrial receptivity (Table 4 and Fig. 1). However, when the endometrium is too thick, pregnancy rate may decrease [23, 24]. In addition, more adequate downregulation caused lower E2 levels on the day of trigger and reduced the negative impact of hypoestrogenemia on endometrial receptivity in the EFLL group, compared to the MLSL group [25, 26]. The conversion to freeze all cycles due to OHSS was also reduced, allowing patients in the EFLL group more opportunities to have a fresh transfer cycle.

This study showed that the ectopic pregnancy rate was lower in the EFLL group than in the MLSL group. Logistic regression suggested that ovulation induction protocols and endometrial thickness on the day of trigger were independent factors for ectopic pregnancy. Endometrial thickness reflects the receptivity of the endometrium [21], and the endometrial thickness on the day of trigger was significantly lower in the ectopic pregnancy group than in the non-ectopic pregnancy group. The increased endometrial thickness in the EFLL group, compared to the MLSL group, negated the negative impact of lower E2 levels on endometrial receptivity during ovulation induction [25], this led to fewer ectopic pregnancies in the EFLL group than in the MLSL group. In addition, some studies [27, 28] suggested that there was a relationship between the number of transferred embryos and the ectopic pregnancy rate when ART was applied, but after multivariate logistic regression correction in this study, the number of transferred embryos was still not statistically significant for the occurrence of ectopic pregnancy. Our findings were consistent with the study by Ribic-Pucelj et al. [29].

The results of the logistic regression in this study suggest that in addition to pre-pregnancy BMI and fasting plasma glucose, which are important risk factors of GDM in patients with PCOS [30], the ovulation induction protocol was found to be an independent risk factor for GDM for the first time (Supplementary Table S1. and Supplementary Figure S1.). The difference in the occurrence of GDM between the protocols may be attributed to the different drugs and doses applied during ovulation induction. During ovulation induction, the use of long-acting GnRH-a may lead to glucose intolerance and increased insulin resistance [31], resulting in a corresponding increased risk of GDM in the EFLL group. It has been shown that the use of Gn is an independent risk factor for GDM, and after adjusting for confounders, the Gn dose is significantly positively correlated with the incidence of GDM [32]. For the EFLL group, the greater downregulation and lower dose of Gn initiation resulted in a longer time of administration and an increased total dose of Gn used during ovulation induction compared to the MLSL group, which may be one of the reasons for the increased incidence of GDM in the EFLL group.

This study comprehensively analyzed the clinical outcomes, perinatal and maternal complications, and fetal outcomes of patients with PCOS using EFLL and MLSL. We found that EFLL is superior to MLSL in terms of fresh embryo transfer rate and clinical pregnancy. Ovulation induction protocol is an independent factor of ectopic pregnancy and GDM. The ectopic pregnancy rate in EFLL is lower than that in MLSL. The incidence of GDM is higher than that in MLSL. However, there are no significant differences in the incidence of preeclampsia, premature rupture of membranes, placenta previa, fetal malformation, neonatal congenital heart disease, and neonatal weight between the two groups.

The strength of this study lies in the comprehensive analysis of perinatal and maternal complications and fetal outcome measures and the analysis of data from a large sample adjusted for potential confounders. At the same time, our study also has some limitations. Our study is a retrospective study and does not consider all confounding factors. Second, our study population only included women with PCOS, who already have a higher risk of gestational diabetes and pregnancy-related complications than the normal population. Therefore, it is still necessary to perform a large, multicenter, prospective study in other populations to verify our conclusion.

## Conclusions

In conclusion, there was a higher fresh embryo transfer rate, clinical pregnancy rate, and live birth rate and lower ectopic pregnancy rate for patients with PCOS who underwent EFLL compared to those who underwent MLSL. This indicates that EFLL can be used as an ideal ART therapy for PCOS patients, but for obese patients, weight loss prior to assisted reproduction should be encouraged to reduce the risk of GDM.

## Supplementary Information

**Additional file 1: Supplementary Table 1.** Univariate logistic regression performed for GDM. **Supplementary Figure 1.** Forest plot: multivariate logistic regression performed for GDM.

## Data Availability

The data sets used or analyzed during the current study are available from the corresponding author on reasonable request.

## Abbreviations

EFLL: Early-follicular long-acting GnRH agonist long protocol
GnRH-a: Gonadotropin-releasing hormone agonist
IVF: In vitro fertilization
ICSI: Intracytoplasmic sperm injection
MLSL: Midluteal short-acting GnRH-a long protocol
PCOS: Polycystic ovary syndrome
