# Supplementary material for: Is early-follicular long-acting GnRH agonist protocol an alternative for patients with polycystic ovary syndrome undergoing in vitro fertilization?
Source: Reprod Biol Endocrinol. 2022 Sep 10;20:137. doi: 10.1186/s12958-022-01007-z (PMC9463774; doi:10.1186/s12958-022-01007-z)
Supplement: Supplementary file 1 — Additional file 1: Supplementary Table 1. Univariate logistic regression performed for GDM. Supplementary Figure 1. Forest plot: multivariate logistic regression performed for GDM. [file 12958_2022_1007_MOESM1_ESM.docx]

***Supplementary Table 1.*** Univariate logistic regression performed for GDM

|  | non-GDM | GDM | OR（95%CI） | P value |
| --- | --- | --- | --- | --- |
| Age | 28.680±3.652 | 29.780±4.295 | 1.079 (1.010,1.153) | 0.026 |
| BMI | 24.191±3.526 | 25.996±3.381 | 1.151 (1.070,1.237) | 0.001 |
| FPG | 4.836±0.478 | 5.219±0.575 | 3.865 (2.423,6.167) | 0.001 |
| Fertilization method |  |  |  |  |
| IVF | 1077/1356 (79.42) | 49/59 (83.05) | Reference | - |
| ICSI | 279/1356 (20.58) | 10/59 (16.95) | 1.269 (0.635,2.538) | 0.500 |
| Treatment |  |  |  |  |
| MLSL | 347/1356 (25.59) | 5/59 (8.47) | Reference | - |
| EFLL | 1009/1356 (74.41) | 54/59 (91.53) | 3.714 (1.474,9.360) | 0.005 |
| Gravidity | 0.480±0.848 | 0.510±0.751 | 1.033 (0.765,1.395) | 0.813 |
| No. of embryos transferred | 1.740±0.453 | 1.710±0.457 | 0.892 (0.507,1.568) | 0.691 |
| No. of gestational sac | 1.31±0.465 | 1.29±0.457 | 0.913 (0.515,1.618) | 0.755 |
| Multiple pregnancy | 365/1356 (26.92) | 14/59 (23.73) | 0.845 (0.458,1.557) | 0.589 |

***Supplementary Figure 1. Forest plot：***multivariate logistic regression performed for GDM


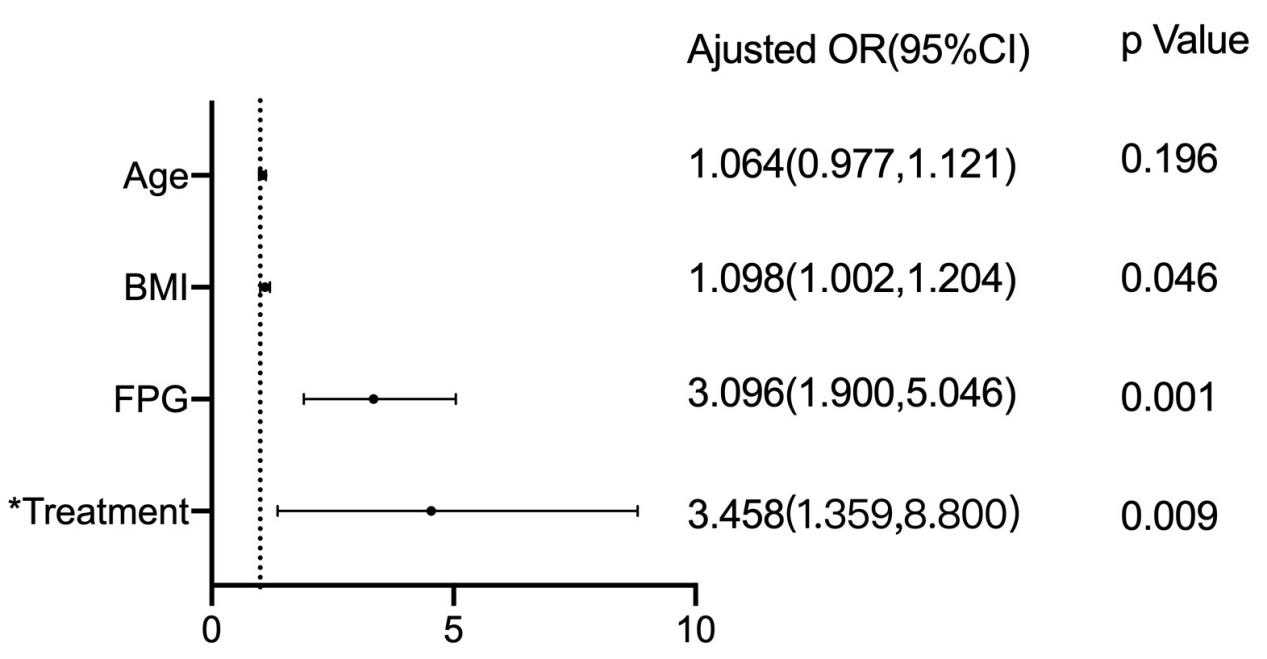


Note: Age, pre-pregnancy BMI, FPG and treatment were included for the multivariant logistic model for GDM.

*MLSL used as a control.
